# Evaluating Common De-Identification Heuristics for Personal Health Information

**DOI:** 10.2196/jmir.8.4.e28

**Published:** 2006-11-21

**Authors:** Khaled El Emam, Sam Jabbouri, Scott Sams, Youenn Drouet, Michael Power

**Affiliations:** ^5^Gowling Lafleur Henderson LLPOttawaONCanada; ^4^Departement d’Informatique et de StatistiqueFaculte de Sciences Economiques et de GestionUniversite Lumiere Lyon 2LyonFrance; ^3^Department of Geography and EnvironmentLondon School of Economics and Political ScienceLondonUK; ^2^School of Computer ScienceCarleton UniversityOttawaONCanada; ^1^University of Ottawa and CHEO Research InstituteOttawaONCanada

**Keywords:** Privacy, confidentiality, HIPAA, security, data disclosure, ethics

## Abstract

**Background:**

With the growing adoption of electronic medical records, there are increasing demands for the use of this electronic clinical data in observational research. A frequent ethics board requirement for such secondary use of personal health information in observational research is that the data be de-identified. De-identification heuristics are provided in the Health Insurance Portability and Accountability Act Privacy Rule, funding agency and professional association privacy guidelines, and common practice.

**Objective:**

The aim of the study was to evaluate whether the re-identification risks due to record linkage are sufficiently low when following common de-identification heuristics and whether the risk is stable across sample sizes and data sets.

**Methods:**

Two methods were followed to construct identification data sets. Re-identification attacks were simulated on these. For each data set we varied the sample size down to 30 individuals, and for each sample size evaluated the risk of re-identification for all combinations of quasi-identifiers. The combinations of quasi-identifiers that were low risk more than 50% of the time were considered stable.

**Results:**

The identification data sets we were able to construct were the list of all physicians and the list of all lawyers registered in Ontario, using 1% sampling fractions. The quasi-identifiers of region, gender, and year of birth were found to be low risk more than 50% of the time across both data sets. The combination of gender and region was also found to be low risk more than 50% of the time. We were not able to create an identification data set for the whole population.

**Conclusions:**

Existing Canadian federal and provincial privacy laws help explain why it is difficult to create an identification data set for the whole population. That such examples of high re-identification risk exist for mainstream professions makes a strong case for not disclosing the high-risk variables and their combinations identified here. For professional subpopulations with published membership lists, many variables often needed by researchers would have to be excluded or generalized to ensure consistently low re-identification risk. Data custodians and researchers need to consider other statistical disclosure techniques for protecting privacy.

## Introduction

The adoption of electronic medical records (EMRs) is growing [1-5]. Researchers are increasingly turning to EMRs as a source of clinically relevant patient data. There are calls for EMRs to support secondary uses of this data for observational studies, such as epidemiologic and health services research [6]. On the other hand, a majority of patients, and the public in general, are concerned about unauthorized disclosure and use of their personal health information in an era of the EMR [7-11]. Furthermore, rates of medical identity theft have been increasing, and the risks are exacerbated with the use of EMRs [12].

Epidemiologic and health services research commonly proceeds without express consent from subjects. There are good reasons for this. It has been shown that requiring consent introduces biases in recruitment because those individuals who do not consent or who are difficult or impossible to request express consent from tend to be different on important characteristics than those who consent and are actually recruited. In some cases, the express consent requirements also increase the cost and duration of the research [13-25].

Excessive restrictions on researchers’ access to identifiable health information is considered detrimental to society at large because many beneficial studies can not be done [26,27].

To safeguard privacy, often one of the requirements for waiving express consent is that the data be de-identified at the earliest opportunity [28]. This is important because there is evidence that individuals can be re-identified using common variables (such as zip code, date of birth, and gender) by linking to publicly available information [29,30]. In addition, identifiability is a key consideration for institutional research boards in deciding whether consent is required [31].

There are different methods for de-identification: statistical disclosure control (SDC) methods [32] and heuristic methods. In practice, SDC methods are not used that often [28,33]; therefore, we focus on heuristic methods. A heuristic approach to de-identification consists of rules about which variables to generalize (also known as aggregation) and which variables to exclude from a data set when it is disclosed. For example, under the US Health Insurance Portability and Accountability Act (HIPAA), two of the three de-identification methods stipulated in the Privacy Rule require the removal of potential identifying variables as defined in the Safe Harbor List and the Limited Data Set [34]. The Canadian Institutes for Health Research privacy guidelines provide examples of generalizing variables (eg, generalizing date of birth to age and generalizing geographic information) as a means to reduce identifiability [35]. Clinical researchers often follow heuristics to ensure that the data they collect and disclose are anonymized, for example, some assume that using initials and date of birth to identify subjects poses low risk of re-identification by those not involved in their study [28]. Various de-identification heuristics are used to decide which variables to exclude when pharmacy prescription records are released to commercial data aggregators [36].

De-identification by removing or generalizing variables from a data set necessarily results in loss of information and may hinder drawing accurate conclusions from that data [37]. The amount and criticality of that loss will depend on the specifics of the data set and the questions the data set is intended to answer. But most researchers would argue that variables, such as date of birth (or its generalization to age) and gender, are critical for many analyses, and geographic information (such as zip/postal codes) may also be necessary [38,39].

Given that there is potentially a high cost to using de-identification heuristics, it is essential to determine whether common de-identification heuristics used in practice today do indeed ensure that the risks of re-identification are low. If they do ensure low re-identification risk, then a case can be made for complying with these heuristics. If there is evidence that they do not ensure low re-identification risk, then the research community needs to consider alternative SDC methods as a means to de-identify data sets and reduce the need for excluding or generalizing important variables.

In this paper we evaluate whether common de-identification heuristics ensure a low level of re-identification risk across different data sets and sample sizes (since the risk of re-identification varies with sample size [32]). The common heuristics we evaluate are a union of a subset defined in the HIPAA Privacy Rule, currently practised in clinical research, and presented in privacy guidelines. If the heuristics ensure a consistently low level of risk, then one can have confidence in using them to de-identify any data set.

### Categorization of Variables

It is useful to categorize variables in a research data set into the following set of mutually exclusive categories since each category is treated differently in the context of de-identification:

- **Identifying variables.** These are variables that can directly identify individuals, such as name, email address, telephone number, home address, social insurance number, and medical card number. Since these variables are obvious identifiers, if they are included, the data set is clearly not de-identified. In some cases, more than one identifying variable is needed to identify an individual uniquely. For example, the name “John Smith” appears 298 times in a search of the public telephone directory in Ontario. However, combined with a telephone number, the individual can be more easily identified uniquely.
- **Quasi-identifiers.** These are variables that do not directly identify an individual but can play an important role in indirect re-identification. One way in which quasi-identifiers can be used for re-identification is by linking to external databases containing identifying variables (record linkage). There are some quasi-identifiers that have been studied more extensively than others, such as gender, date of birth, and postal/zip code.
- **Nonidentifying variables.** Such variables may be, for instance, clinical and lab values. They are generally not useful for re-identification. For example, an indicator variable on whether an individual has pollen allergies would most likely be a nonidentifying variable.

It is common in disclosures of health data sets that the identifying variables are removed. We will therefore focus on risks from the quasi-identifiers.

### Uniqueness and Re-identification

Uniqueness of individuals in a data set will have an impact on the risk of re-identification. We assume that a data set is a sample from some population. If an individual has a unique combination of values for the quasi-identifiers among other members of the population, then that person is *population unique*. If an individual has a unique combination of values for the quasi-identifiers among other individuals in the sample, then the individual is *sample unique*. If an individual is population unique, then, by definition, that person is sample unique, but not vice versa.

Uniqueness makes re-identification more likely through two common mechanisms: traceability and record linkage, which are explained below.

If a person is easy to trace in the real world, then that increases re-identification risk. For example, let’s say that there are two quasi-identifiers in a data set: city/town and profession. If an individual has the values “Ottawa” and “Mayor,” then it would be relatively easy to figure out who that individual is, even if there is no identifying information in the record.

**Figure 1:**
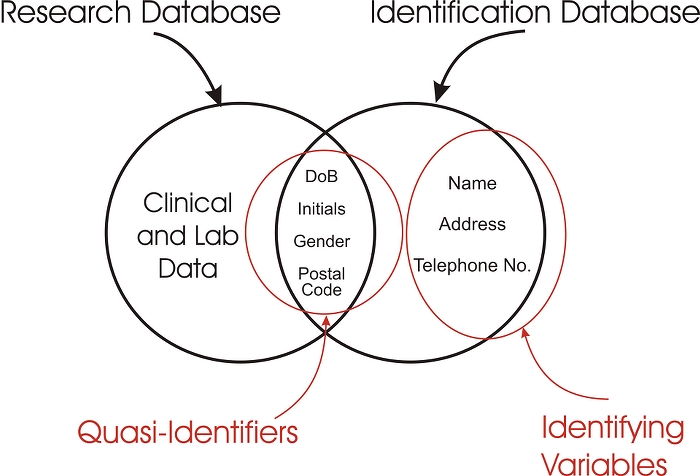
Illustration of record linkage of a research database and another identification database

If a particular set of quasi-identifiers in a record can be linked with a record in another database to re-identify individuals, then it can be said that the risk of re-identification is high. This is illustrated in Figure 1. Let us assume an individual (say, a researcher) has a de-identified research database containing some clinical data and that this database also contains quasi-identifiers such as initials, date of birth, gender, and postal code. If we could get access to an identification database or construct one from public data sources, with the same four variables as the research database as well as identifying variables such as name, address, and telephone number, then it would be possible to link the two databases and re-identify the individuals in the research database.

This means that if someone has access to a research database containing these quasi-identifiers, then it would be possible to re-identify the subjects by performing the record linkage with an appropriately constructed identification database. In principle, an identification database can be constructed in a number of ways:

- publicly available information from government bodies and professional associations
- data already available to an intruder from other sources, for example, a researcher with data available from another project (We will use the term “intruder” here for convenience, but it is recognized that re-identification may have legitimate purposes as well.)
- the circle of acquaintances of the intruder, which is the set of individuals from the population about which the intruder knows the values of the quasi-identifiers
- commercial organizations that sell databases containing data on members of the general public
- mining the Internet for information that individuals post about themselves (eg, resumes or personal Web pages) [40,41]
- inadvertent access to data, such as the purchase of surplus or second-hand computer equipment with data remaining [42]
- illegal activities, such as theft of computers with data or theft of unencrypted backup tapes during transit

**Figure 2:**
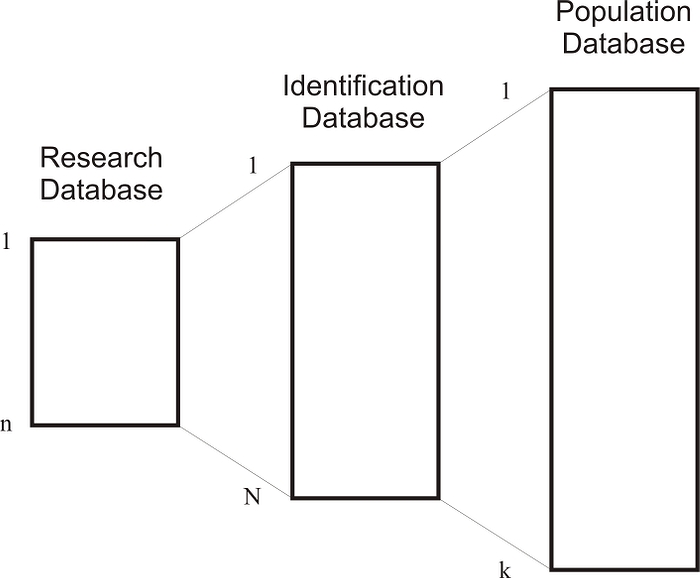
The relationship between the research database, the identification database, and a hypothetical population database

Only the individuals in the identification database can be re-identified. If the identification database has all of the population of individuals in it (ie, *N* = *k* in Figure 2), then all members of the population are potentially re-identifiable. The research database would represent a sample from the identification database, with *n* < *N* in Figure 2. In the scenarios we are considering, an intruder is attempting to re-identify all the individuals in the research database.

Traceability and record linkage are two different things, although the underlying property (uniqueness) is the same, and one does not imply the other. For example, if we have a physician with a date of birth 1 January 1950 and that date is unique among all physicians in a province (ie, it is a population unique value), then that individual would still be difficult for an intruder to trace among the population of physicians. However, if an identification database of all physicians exists and the date of birth is one of the variables, then that physician would be easy to re-identify through record linkage.

We are only concerned with re-identification risk due to record linkage. Therefore, an important requirement is that an intruder is able to create an identification database. Only the quasi-identifiers that can exist in an identification database are relevant.

### Commonly Used Quasi-identifiers

In the following paragraphs, we consider some of the commonly used quasi-identifiers in clinical research and their generalizations.

The first set of quasi-identifiers is defined in HIPAA. The HIPAA Privacy Rule defines three methods to de-identify a data set; two of these provide a very specific set of variables that should not be included in the data set for it to be considered de-identified. Both list a number of identifying variables and quasi-identifiers. We are only concerned with the quasi-identifiers. In the Safe Harbor method, two types of quasi-identifiers must be excluded:

- all geographic subdivisions smaller than a state (except the initial three digits of a zip code if the population in that zip code is more than 20000)
- all elements of dates (except year) or dates relating to an individual, including date of birth

The Limited Data Set method allows dates and excludes only the street address from the geographic information.

There is evidence that clinical researchers in Canada follow the HIPAA guidelines since these provide more precise prescriptions than anything else available locally [28]. Previous studies have performed successful matching experiments using date of birth, gender,and zip code as quasi-identifiers [29,30]. A recent qualitative study found that researchers use a combination of initials and date of birth to identify subjects [28]. Guidelines for protecting the privacy of personal information often include date of birth and geographic information as risky variables [35,43].

A generalization schedule for the geographic and date of birth information is as follows [35] (customized to a Canadian context):

- full postal code >> forward sortation area (first three digits of the postal code) >> city >> region (first character of the postal code)
- date of birth >> year and month of birth >> year of birth

A list of the quasi-identifiers extracted from the literature and evaluated in our study are given in Textbox 1.

**List of nine quasi-identifiers extracted from the literature**

**Table:** 

date of dirth (DoB)	forward sortation area
DoB – month and year	city
year of birth	region
gender	initials
postal code	

## Methods

The objective was to evaluate re-identification risk for common quasi-identifiers and their combinations. The research method consisted of two steps:

1. constructing multiple identification databases
2. evaluating re-identification risk and its stability across data sets and sample sizes

### Constructing Identification Databases

While there have been re-identification experiments in other nations, such as the United States [29,30], the United Kingdom [44], and Germany [45], there have been no attempts to construct identification databases in Canada. We therefore first attempted to construct identification databases using public sources in the province of Ontario.

### Identifying Data Sources

Multiple sources of public data were sought as described below. Public data are defined as data that are available to the general public for free or a reasonable fee, with a reasonable amount of effort to access them, and without a review by the data holding institution or the need to sign a confidentiality or data sharing agreement with the data holding institution that restricts what can be done with the data.

All 29 Ontario government ministries were contacted. We identified staff in the freedom of information and privacy (FOIP) office in each ministry, if one existed. In all ministries except one, the FOIP office was contacted and we conducted a telephone interview with at least one staff member about the data that they release and the procedures for us to get that data.

A sample of commercial information brokers in Canada claiming to sell population databases were contacted to determine the type of data they hold, the sources of data, how the databases they sell were constructed from the sources, and conditions of disclosure. After examination of their websites, we followed up with phone calls to verify the information and get additional details. These brokers included Americanada, Prospects Influential, Nation Reach, and InfoCanada.

Sources of genealogical data were examined as well. These include data available through the Ottawa Public Library and the National Archive Centre. These include birth, baptism, death, marriage, adoption, and divorce data. Both of these locations were visited and staff on site were interviewed to determine the types of data available and how those data were released.

Professional societies frequently release comprehensive member lists. In some instances, work addresses and gender are also provided. We contacted a sample consisting of the College of Physicians and Surgeons of Ontario (CPSO), Law Society of Upper Canada (LSUC), Professional Engineers Ontario, College of Physiotherapists of Ontario, and the College of Occupational Therapists of Ontario. For all these professional societies, the membership lists were available on the Web. Commercial brokers, such as LexisNexis, WestLists, LawyerLocate, and Martindale, also provided lists of professionals. For commercial organizations, the data holdings were advertised on the websites. We followed up with phone calls to ensure the accuracy of the information on the Web and to fill in any missing details in our understanding of their data holdings.

We also contacted Statistics Canada and examined the information in the various products from the 2001 census data set. In particular, we focused on tabulations giving gender and age, and on microdata releases. Additionally, we contacted Elections Canada and interviewed volunteers in election campaigns to understand how voter lists are used.

### Creating Identification Databases

An identification database consists of two elements: quasi-identifiers and identifying information. There are two general methods that can be used for constructing an identification database:

- **Direct method.** A public source will have both elements needed for an identification database. An example would be a voters list.
- **Indirect method.** We first find a source with the identifying information on individuals, and then these are linked with another source that contains the quasi-identifiers.

We followed both methods to create an identification database.

### Evaluating De-identification Heuristics

#### Measuring the Risk of Re-identification

The measure of the risk of re-identification we used is grounded in the matching process that an intruder would likely use in order to re-identify a de-identified data set. Our measure of re-identification risk assumes that an intruder is attempting to re-identify all of the individuals in the research database by matching the individuals in the research database with records in an identification database using the quasi-identifiers. We predict the probability that a randomly selected individual can be matched successfully. Because only those individuals in the identification database can be re-identified, we assume that the identification database represents the population and the research database is a sample from that population (ie, only a subset of the individuals would be in the research database).

The estimation method used was data intrusion simulation (DIS) [46,47]. This predicts the risk of re-identification using this particular attack scenario (other attack scenarios are discussed later in the paper). DIS predicts the conditional probability that a unique match of a record in the identification database with a record in the research database is a correct match:

*P(correct match|unique match) = P(cm|um)*.

**Figure 3:**
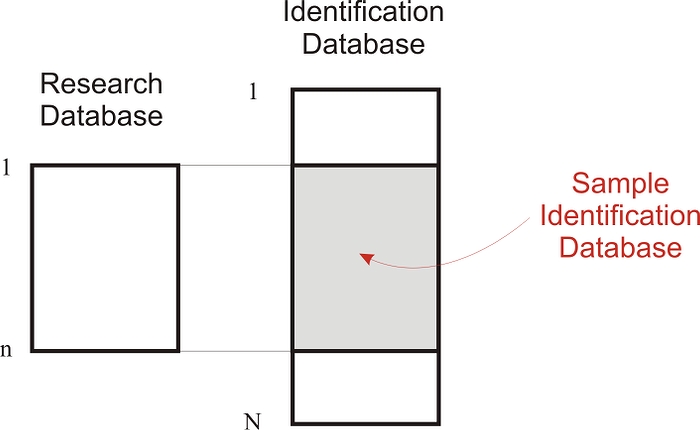
A sample identification database (shown shaded) for data intrusion simulation

It should be noted that we do not actually need a complete research database or a complete identification database to estimate re-identification risk. All that is needed is a sample identification database, as shown in Figure 3, containing only the quasi-identifiers and identifying variables for the *n* individuals in the research database. No actual clinical or lab data are required to perform the risk analysis.

A Monte Carlo simulation, described in Appendix 1, illustrates the robust performance of DIS under a range of sampling fractions. Other measures of re-identification risk that have been proposed do not produce accurate results for small sampling fractions and are not specific to a type of attack [48,49].

Although there are no generally accepted re-identification thresholds, one can easily make the case that any probability of a successful attack greater than 0.01 would be unacceptable (for a large database, a probability of successful attack as high as 0.01 would compromise the privacy of a relatively large number of individuals). We therefore use that as a threshold for interpreting the risk results.

#### Evaluating the Heuristics

In our evaluation, three parameters were varied: the data set, the sample size, and the quasi-identifier combinations evaluated.

1. We constructed two identification databases to see whether the risk findings carried across them.
2. For each combination of quasi-identifiers, we decremented the sample size by one observation, chosen at random from *n* to 30, and determined whether *P(cm|um)* was below the threshold at the reduced sample size. This process was iterated 100 times for each sample size, and the average number of times that the risk was below the threshold was taken as the result for that sample size. If the risk was below the threshold, then we considered the quasi-identifier combination as “safe” (ie, one that ensures low re-identification risk quite often). We then looked at the frequency of quasi-identifiers that were considered “safe” across all sample sizes. If a quasi-identifier was “safe” more than 50% of the time, then it ensured that the risk was below the threshold across sample sizes.
3. We considered all possible individual and 2-, 3-, and 4-fold combinations of different quasi-identifiers.

## Results

### Constructing an Identification Database

#### Direct Method

The privacy offices at government ministries do provide oversight on the release of data. However, they are unable to control all possible releases and therefore only intervene when there is a complaint, an access to information request, or when they are asked for assistance from one of the departments. None of the privacy offices were able to produce a basic listing, even approximate, of all personal data releases from their ministry.

The commercial information brokers we contacted linked publicly available Statistics Canada census data with telephone directory data. Because of the aggregations performed on census data that are released, information such as age is only approximate. In addition, these would still not be population databases because not everyone has a telephone registered in their name. A recent independent study has confirmed that this is the approach used when commercial brokers utilize public data [25].

Birth and death notices are available from the General Registrar of Ontario. However, it is necessary to prove a relationship to the individual about whom data are being requested in order to get access to that information. Driver licence information also requires the name and the driver’s licence number in advance to be able to make an information search request. Therefore, in both of these cases, it is not possible to construct a database for record linkage.

Voter lists are made available to candidates or their party representatives. These lists include the name, address, and date of birth of eligible voters. That information is to be used solely for the purposes of an election, including raising funds. Party members participating in an election campaign are bound by the party oath in terms of protecting that information. Volunteers on election campaigns who are not party members are not bound by an oath and would not normally sign a confidentiality agreement. Therefore, there are ways to get the voter list for a re-identification attack, but that would require deceptive practices and such use would likely go against the Elections Act.

Some commercial brokers may collect data sets directly from the public through surveys or subscription lists, or they may purchase these from retailers (eg, loyalty card users or warranty card information). These data sets may contain the quasi-identifiers we are interested in as well as identifying information. However, these do not include all members of the population.

We were therefore unable to construct an identification database for the whole population using the direct method.

#### Indirect Method

We were able to construct an identification database using the indirect method. However, it was not possible to do so for the whole population, but only for professional subpopulations, namely physicians and lawyers in Ontario. The list of physicians is published by the College of Physicians and Surgeons of Ontario (CPSO), and the list of lawyers is published by the Law Society of Upper Canada (LSUC).

**Figure 4:**
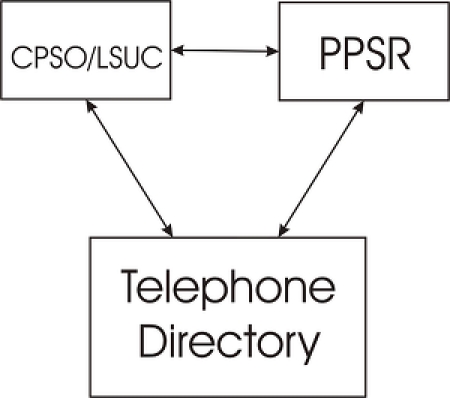
The three main source databases used to construct an identification database for a professional subpopulation

It is possible to link the information in the list (which includes name, practice/firm address, and gender) with the Ministry of Government Services’ Personal Property Security Registration (PPSR) data and the Canada 411 telephone directory data (both available on the Internet, the former for a fee) to identify the home postal code and date of birth (Figure 4).

We created a random sample data set of 236 physicians and 189 lawyers across Ontario with the quasi-identifiers under study. This represents a 1% sampling fraction of all registered physicians who are still active and practising in Ontario (23506) and all practising lawyers in Ontario (18728). The variables in our identification database were full name, gender, graduation date (CPSO only), date of birth, address for place of work (practice/firm), home address, and home telephone number.

**Table 1:** Ability to get various data elements on physicians and lawyers, with the source of the data (n = 236 for CPSO; n = 189 for LSUC)

**Quasi-identifier**	**CPSO (%)**	**LSUC(%)**
home postal codes (source: PPSR and telephone directory)	60	45
practice/firm postal codes (source: CPSO/LSUC)	100	100
date of birth (source: PPSR)	40	45
gender (source: CPSO/genderizer for LSUC data)	100	100
initials (source: CPSO/LSUC)	100	100

Table 1 shows the success rates in getting the quasi-identifiers for an identification database. Name (and initials), practice postal codes, and gender are available from the CPSO. Therefore, we can obtain these for all physicians. Name and firm postal codes are available from LSUC. Since the LSUC does not publish gender in their public listing, genderizing software (see the analysis of the accuracy of such tools in Appendix 2) was used to estimate gender for the lawyers from their first names. We were able to determine the home postal code and date of birth from the PPSR for both professions. Additional verification of identity and home postal code was performed by checking against the Canada 411 website (online telephone directory). To verify that matches were correct, we also consulted the land registry in some instances to confirm addresses. Records were flagged for additional manual investigation under two conditions: (1) if the distance between the work and home postal codes was more than 100 km (determined by calculating the Euclidean distance), and (2) if, for physicians, the graduation date and date of birth were less than 25 years apart.

 As evident in Table 1, it was not always possible to get the date of birth (40% and 45% success rates for physicians and lawyers, respectively) and the home postal code (60% and 45% success rates for physicians and lawyers, respectively). There was also a gender difference. We were able to get the home postal code for 49% of all female physicians vs 63% for males, the date of birth for 29% of all female physicians vs 45% for males, the home postal code for 40% of all female lawyers vs 48% for males, and the date of birth for 40% of all female lawyers vs 48% for males.

#### Stability of Heuristics Across Sample Sizes and Data Sets

Table 2 provides the results of the stability analysis. The table shows the percentage of times that a particular combination of quasi-identifiers was found to be “safe” (ie, below the 0.01 risk threshold) as we varied the sample sizes across the two data sets. In total, 143 quasi-identifier combinations were evaluated. We only show those quasi-identifiers and their combinations that had percentages higher than 50%. If a quasi-identifier is not “safe” at least 50% of the time, then we can make the case that it is not stable. This means that if the quasi-identifier combination was above the risk threshold more than 50% of the time, it was therefore sensitive to sample size.

The findings indicate that gender, region, and year of birth are individually all relatively stable across sample sizes and data sets, as well as the combination of region and gender. This means that the inclusion of these quasi-identifiers in a released data set does not increase the risk of re-identification.

The gender and year of birth combination was low risk 80% of the time only for the CPSO data set. Consequently, we consider it unstable across data sets.

**Table 2:** Percentage of time a quasi-identifier or combination of quasi-identifiers was considered “safe” more than 50% of the time (as sample sizes were varied from 30 to the maximum)

**Safe Quasi-identifier or Combination**	**Percentage of Time Quasi-Identifiers Were Below the Threshold**	
	**CPSO (%)**	**LSUC (%)**
gender	100	100
region	93	65
DOB – year	94	85
gender + region	85	82
gender + DOB – year	80	–

## Discussion

### The Stable De-identification Heuristics

We found that only a small subset of the quasi-identifiers represented a consistently low risk of re-identification across both sample size changes and data set changes. Most quasi-identifiers (including generalizations) were not stable. In terms of formulating heuristics for the de-identification of data, the following quasi-identifiers were low risk (out of the set that we evaluated):

- region alone
- gender alone
- year of birth alone
- combination of gender and region

A corollary of this result is that all other individual quasi-identifiers and all other combinations are not safe.

### Constructing Identification Databases

An important prerequisite for a record linkage attack is the ability to construct an identification database. It was possible to do so for professionals whose associations publish their membership lists. We found that it is more difficult to construct an identification database for adult females. It would also not be possible to perform a similar exercise on youth because youth would not have any loans that are registered, would not have property registered in their names, and would not have telephone numbers in their names. Therefore, their names would not appear in any of the publicly available data sources that we investigated. Also, it would not be possible to do so for professional associations that do not publish their membership lists.

We found that it is not possible to construct an identification database for the whole population of Ontario. We were unable to do so using public sources, with either the direct or indirect method. In Canada, the ability of researchers to access and use information is qualified by legislative restrictions designed to protect the privacy of individuals. This information may consist of what otherwise may be considered in other countries as “public data” (eg, driver’s licence databases or public information).

In some instances, population databases are available for access but have certain data elements removed. For example, in Ontario, personal information is collected by the Ministry of Transportation under the authority of section 205 of the Highway Traffic Act. The information forms part of a public record and is used for the administration of the Ministry’s driver, vehicle, and carrier programs. However, while residence address information is collected, it is not considered part of the public record and is not available to the general public. A further qualification is that only “authorized” requestors who have been approved and have entered into a contractual agreement with the Ministry may obtain residence address information for certain limited purposes. These purposes do include research by educational or research organizations. This limited degree of access is safeguarded by application of public sector privacy legislation in Ontario—the Freedom of Information and Protection of Privacy Act. The federal government and each of the 13 provincial/territorial jurisdictions in Canada have similar legislation designed to protect the privacy of individuals and protect personal information held by government bodies.

Under such laws, “personal information” is broadly defined to generally mean recorded information about an identifiable individual, including “any identifying number, symbol or other particular assigned to the individual.” Once it has been determined that a record contains personal information, these types of statutes generally prohibit the disclosure of this information, except in certain circumstances. One instance where disclosure may occur is when “personal information [is] collected and maintained specifically for the purpose of creating a record available to the general public,” which is the case with the PPSR database we used.

The preceding discussion was directed to government holdings of information. The use of publicly available information held by non–public sector entities is governed by private sector privacy legislation that exists in Canada. At the federal level and in those jurisdictions that do not have comprehensive personal information protection statutes, the legislation in question is the Personal Information Protection and Electronic Documents Act*.* British Columbia, Alberta, and Quebec have their own statutes that place restrictions on the collection, use, and disclosure of personal information by non–public sector entities.

Generally, the provincial statutes governing non–public sector entities apply to publicly available information, making the use of such information subject to a consent requirement. Use without consent is permitted for certain prescribed sources of information. The federal statute permits the collection, use, and disclosure of publicly available information but then defines “publicly available information” by regulation. This include names, addresses, and telephone numbers in a telephone directory; name, title, address, and telephone number that appear in a professional or business directory available to the public; and personal information that appears in a registry collected under a statutory authority.

### Generalization of Findings

Our data sets were constructed for an Ontario population. We have investigated the ability to construct similar identification databases in Canada. The two main data sources were the PPSR and telephone directory. There is an online telephone directory for every province. In Appendix 3, we have listed the PPSR sources for all provinces and territories. These would allow the construction of similar identification databases holding similar types of quasi-identifiers.

The risk of re-identification due to record linkage is affected by population uniqueness. For example, if we considered another profession that was heavily skewed toward males (say, underwater welders), then a female underwater welder is likely to be population unique. In that case, gender would not be a “safe” quasi-identifier. On the other hand, if there were no female underwater welders at all, then gender would be “safe.” Notwithstanding such variations, our results provide concrete evidence that many common quasi-identifiers are high risk for some professions. That such examples exist for two mainstream professions makes a strong case already that the high-risk quasi-identifiers and combinations should not be disclosed.

As noted above, Canada has relatively strict privacy laws that restrict the amount of information about individuals that is disclosed and available for use in the public domain. Consequently, we expect that, from the perspective of re-identification risk, other jurisdictions with less restrictive laws would likely have higher risks of re-identification and more “unsafe” quasi-identifier combinations. Therefore, our list of “unsafe” quasi-identifiers is likely smaller than what one would find in a less restrictive jurisdiction (in terms of availability of information through public sources).

Given that the risk is affected by the ability to construct an identification database, this study can serve as a template for other jurisdictions to perform a risk assessment.

### Managing the Risk of Re-identification

There are two ways to manage the risk of re-identification due to record linkage: exert control on the quasi-identifiers that are included in a research database, and exert control on the ability to create an identification database.

The first approach is simple to implement in practice. However, the quasi-identifiers that were found to be high risk constitute variables that would be considered important in many observational studies. These results highlight the unsatisfactory consequence of basing de-identification practices on such heuristics. This suggests that data custodians should consider using more sophisticated statistical disclosure control techniques [32] rather than basic heuristics about which variables to exclude and generalize. With such methods, it would be possible to retain important variables but at the same time reduce the risk of re-identification. This suggestion is essentially the third method defined in the HIPAA Privacy Rule for de-identifying data sets.

Two approaches to reduce the likelihood of being able to create an identification database are removing membership lists from the public domain and using financial deterrents.

Professional associations that make their membership lists public should re-evaluate this practice given the privacy consequences of doing so. The fact that such lists exist and are so easily accessible makes it possible to construct identification databases that can be used for launching re-identification attacks through record linkage. The most desirable action is to remove these lists from the public domain. Failing that, one would argue that at least the affected members should be made aware of the risks such disclosure entails.

When releasing membership lists it is also important to ensure that there are no unique values on all combinations of quasi-identifiers in the data set. The released data set would match the population, and population unique values represent a high risk of re-identification. For example, if we wish to release a list of all underwater welders and there is only one female, then that particular record should not be released, or the gender variable should not be released.

Another effective method is to impose fees for access to the registries that are used to create an identification database. Such an access fee would be small enough to be, at most, an inconvenience to most legitimate users, but would represent a prohibitive cost for most intruders. There was a financial deterrent for the registries that were used in this study. At the time the study was conducted, there were 23506 physicians registered in Ontario who were still active and practising in the province. To be able to construct a complete identification database with records containing names, addresses (including postal codes), gender, and date of birth for all physicians in Ontario, it would cost at least Can $188048 because of the PPSR search fee (which is Can $8 per search). Similarly, there were 18728 registered lawyers, making the minimal cost for constructing an identification database Can $149824. While we needed only a 1% sample to estimate risk, an intruder would require a complete identification database for re-identification.

### Limitations

In our study, we used a particular measure of the risk of re-identification. This measure assumes a particular attack scenario on the database. Our conclusions are limited to that attack scenario, but there are other possible scenarios of attack; for example:

- an intruder may already know that an individual exists in a research data set and wishes to identify the record belonging to that individual
- a specific individual or small number of individuals have unique characteristics in a released data set (eg, a specific diagnosis) and an intruder wants to identify these specific individuals in the data set

We did not consider these types of attacks, but they certainly would be important ones to investigate in the future. We also made the assumption that all individuals in the research database have the same probability of re-identification. Future work should consider re-identification risk at the record level. For instance, by knowing which specific records are high risk, they can be targeted for disclosure control actions. This would result in fewer distortions to a data set.

The threshold for high risk that we chose was arbitrary. There are no precedents for defining acceptable risk of re-identification for the release of personal health information; therefore, the risk threshold will have to evolve as our understanding of acceptable risk evolves. Furthermore, acceptable risk is not static. Society may get to accept higher risk in return for specific conveniences or personal benefits. Conversely, acceptable risk may decrease if there is a perception of abuse by custodians or there is a sharp rise in medical identity theft.

There may be a profession whose distribution of quasi-identifiers has many unique observations (eg, predominantly of a single sex or very sparsely distributed geographically). In such a case, the “safe” quasi-identifiers identified here may no longer be safe. Future research should investigate other public membership lists to determine the uniqueness of their quasi-identifier values to test the generalizability of these findings across professions.

### Conclusions

One commonly used approach to protect data that may be disclosed for research purposes is to de-identify it. Specific heuristics for de-identification are included in, for example, the HIPAA Privacy Rule and various privacy guidelines. The heuristics stipulate that variables which present a high risk of re-identification (quasi-identifiers), for example, because they can be used in record-linkage attacks, should be removed or generalized. In this study, we examined such risks by evaluating the re-identification risk due to record linkage with common quasi-identifiers across different data sets and sample sizes.

It was not possible to construct an identification database for the whole population, but it was possible to do so for professional associations that publish their membership lists (eg, physicians and lawyers). Our results indicate that few quasi-identifiers are safe because they maintain the re-identification risk below a threshold. These are region, gender, and year of birth. The only combination that was consistently low risk was region and gender. That such examples of high re-identification risk exist for two mainstream professions already makes a strong case that many common quasi-identifiers should not be disclosed. However, one can also argue that many potentially important variables for researchers would be made unavailable.

These findings therefore indicate that the use of heuristics may be too restrictive and that data custodians should consider more sophisticated statistical disclosure techniques to ensure that important variables are retained in a data set while ensuring that privacy is maintained.

## Figures and Tables

**Figure 5:**
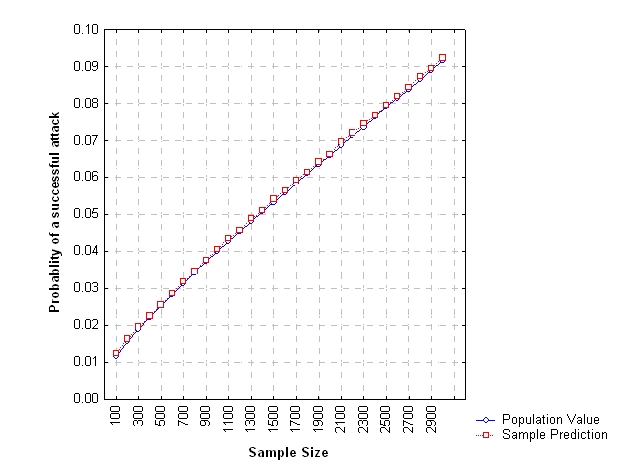
The probability of a successful re-identification attack (y-axis) for various sample sizes (x-axis) based on a Monte Carlo simulation

## Appendix 1

### Monte Carlo DIS Simulation

In this appendix we report on a Monte Carlo simulation to demonstrate and evaluate the characteristics of data intrusion simulation. We used data on the 23506 physicians listed by the College of Physicians and Surgeons of Ontario as our population. We created random samples of various sizes from that population.

For the simulation we drew samples varying in size from 100 to 3000 individuals. A series of three quasi-identifiers were evaluated individually: gender, work postal code, and the work forward sortation area. Each sample size was drawn 1000 times and, in each case, the estimate of the probability of successful re-identification was calculated from the sample. Since we have the population data set as well, it was possible to compute the actual probability using the population data set. The bias of the predicted probability is computed by comparing it with the actual probability.

Figure 1 shows the results for the forward sortation area of the work postal code. The graph shows the predicted and actual values based on the sample and the population, respectively. These are averages across 1000 iterations for each sample size. It can be seen that the bias is quite small for the full range of sampling fractions studied. The highest sampling fraction was just under 13%, and the smallest sampling fraction was 0.04%. The magnitude of bias ranges from 0.00015 to 0.0016. For our purposes, this bias is quite small and indicates that the predicted probability is quite robust even for small sampling fractions. Similar results were obtained for the other quasi-identifiers examined.

It should also be noted that the probability of re-identification increases with sampling fraction. This is consistent with the common recommendation made in the disclosure control community to minimize the size of the sample that is released because that lowers the risk of re-identification. Therefore, one approach to reduce the risk of re-identification is to release a smaller data set.

## Appendix 2

### Evaluating Genderizing Software

The purpose of the evaluation described here is to find out how accurate current software is for predicting gender from first names. This capability is important in the construction of identification databases as a name is often available and we wish to obtain the gender quasi-identifier to match with a research database.

The data set that we tested with was the list of 23506 practising physicians in Ontario, for which we knew the correct gender. A search for genderizing software was performed on MedLine (no date limit), Journal of Marketing (2002 to present), Marketing (January 1996 to present), as well as Web searches on Yahoo and Google. The search terms used were “genderizer or genderizing or genderizing” and “software or tool or API”. Nine products were identified as well as the gender list provided by the US Census Bureau. Of the products, a number of them used the same underlying API. We contacted the vendors for the remaining products and were only able to successfully contact and purchase four products. The Census Bureau list is available for free. Each of the four products as well as the Census Bureau list was used to predict the actual gender for the list of physicians.

The results are shown in Table 1. The overall accuracy shown in the table is the simple proportion of overall predictions that were accurate. Precision, recall, and the f-measure are standard measures of binary classification accuracy. While the accuracy measures are quite high overall and tend to be quite close to each other, Personator with Genderbase had the best results for this data set. This is the tool that we used in our study to predict gender in the lawyer data set (LSUC). Given that this data set consists of heterogeneous Canadian professionals working in an Anglophone environment, it is reasonable to generalize to other similar groups. We cannot make broader generalizations to professionals, for example, in Francophone areas (eg, Quebec).

**Table 3:** Results of evaluating the accuracy of various tools for predicting gender from first names

	**Male**	**Female**
**ParseRat (overall accuracy = 0.81)**		
Precision	0.988	0.989
Recall	0.818	0.80
F-measure	0.89	0.88
	
**Personator (overall accuracy = 0.81)**		
Precision	0.98	0.99
Recall	0.82	0.79
F-measure	0.89	0.88
	
**Personator with Genderbase (overall accuracy = 0.89)**		
Precision	0.98	0.98
Recall	0.9	0.87
F-measure	0.94	0.93
	
**MAILERS+4 (overall accuracy = 0.78)**		
Precision	0.988	0.997
Recall	0.78	0.77
F-measure	0.87	0.87
	
**US Census Bureau (overall accuracy = 0.77)**		
Precision	0.98	0.996
Recall	0.77	0.78
F-measure	0.86	0.88

## Appendix 3

### Personal Property Security Registries

The following is the list of locations across Canada to obtain PPSR information for the construction of identification databases.

**Table:**

**Province/Territory**	**URL**
British Columbia	https://www.bconline.gov.bc.ca
Alberta	Available from authorized registry agents
Saskatchewan	http://www.isc.ca
Manitoba	https://direct.gov.mb.ca/ppr/
Ontario	https://www.personalproperty.gov.on.ca/ppsrweb/en/enquiry/cc_enquiry.jsp
Quebec	http://si2.rdprm.gouv.qc.ca/index.asp
New Brunswick	https://www.web11.snb.ca/snb7001/e/2000/2700e_6.asp
Nova Scotia	http://www.acol.ca/Services/PPR/NS/menu.html
Prince Edward Island	http://www.acol.ca/Services/PPR/PE/menu.html
Newfoundland and Labrador	http://www.acol.ca/Services/PPR/NF/menu.html
Northwest Territories	http://www.acol.ca/Services/PPR/NT/menu.html
Nunavut	http://www.acol.ca/Services/PPR/NU/menu.html

## Abbreviations

CPSO: College of Physicians and Surgeons of Ontario
DIS: data intrusion simulation
EMR: electronic medical record
FOIP: freedom of information and privacy
HIPAA: Health Insurance Portability and Accountability Act
LSUC: Law Society of Upper Canada
PPSR: personal property security registration
